
JOURNAL INFORMATION
==============================
NLM Title Abbreviation: ScientificWorldJournal
Journal ID: ScientificWorldJournal
NLM Journal ID: 101131163
Journal ID: TSWJ

The Scientific World Journal

ISSN: 2356-6140
EISSN: 1537-744X
Publisher: Wiley

ARTICLE INFORMATION
==============================
PMCID: PMC6083995
PMID: 30147581
DOI: 10.1100/tsw.2001.126
Article ID: 273214
Article version: 1
Subjects: Short Report

Control of Cytotoxic Lymphocyte Survival in Vivo By Members of the TNF-R Family

Podack Eckhard R.
University of Miami School of Medicine, Miami, Florida 33101, USA
Publication date: 2001
Electronic publication date: 2001 Sep 24
Volume: 1
First page: 103
Last page: 103
Copyright: Copyright © 2001 Eckhard R. Podack.
Copyright year: 2001
License: This is an open access article distributed under the Creative Commons Attribution License, which permits unrestricted use, distribution, and reproduction in any medium, provided the original work is properly cited.
License URL: https://creativecommons.org/licenses/by/3.0/

==============================
Miami *Nature Biotechnology* Short Reports
*TheScientificWorld* (2001) 1(S3), 103SR
ISSN 1532-2246; DOI 10.1100/TSW.2001.126

### CONTROL OF CYTOTOXIC LYMPHOCYTE SURVIVAL IN VIVO BY MEMBERS OF THE TNF-R FAMILY

Eckhard R. Podack

University of Miami School of Medicine, Miami, Florida 33101

Cytotoxic lymphocytes expressing perforin and granzymes are important effectors for the clearance of viral infections, for immune surveillance and rejection of tumors. They also participate in allogeneic transplant rejection and graft versus host disease. The emergence and survival of cytotoxic CD8+ T lymphocytes (CTL), an important subpopulation among cytotoxic lymphocytes, is carefully regulated both at the level of their generation and their removal. The process is further controlled by the fact that CTL are generated in draining lymph nodes but must exert their effector function at distant sites in tissues (1). Control of effector function therefore must be carefully integrated with CTL migration and apoptosis.

Dramatic expansion of CTL from their precursors can be achieved by secreted heat shock protein gp96-Ig (2) that is recognized by CD91 on dendritic cells, potent antigen presenting cells for T cells. Heat shock protein vaccines cause clonal expansion of cognate TCR transgenic preCTL from a frequency of <1% of all CD8+ cells to a frequency of >20% of the CD8 pool within one week and, upon boosting the response, to >50% of all CD8 cells. This system offers an ideal setting for assessing the homeostatic control of effector CTL pools *in vivo* and measuring their effector cell activity.

Activated CD8+ CTL express numerous receptors and ligands of the TNF-R family. The role of Fas and Fas-L in T cell survival is well documented. Deficiency of either receptor or ligand results in lymphoproliferative disease and autoimmunity, demonstrating a significant function for Fas and Fas-L in terminating clonal expansion and triggering cell removal.

CD8+ CTL of the memory phenotype (CD45RO) upon activation transiently expresses very high levels of CD30, another member of the TNF-R family (3), suggesting an important role for this molecule on CTL. While CD30 is not endowed with a death domain, it can nonetheless signal to FADD via its TRAF binding ability. On the other hand CD30 signals are also able to activate NF-kB and JUNK resulting in proliferation. Recent studies suggest that CD30 signals elicited on cytotoxic lymphocytes may integrate effector function (cytotoxicity), lymphocyte migration and susceptibility to apoptosis. Signals emanating from CD30 up regulated CCR7, down regulated the cytotoxic molecules perforin and granzyme B and affected both pro- and anti-apoptotic molecules. One interpretation of these data suggests that CD30-L expressed in tissues may down regulate effector cell cytotoxicity and redirect CTL towards lymph nodes via the CCR7 receptor (4). Recirculation of CTL to lymph nodes allows reactivation or reprogramming of the effector response, including initiation of apoptosis.

A role for CD30-L in auto immunity induction has been revealed in studies using newly generated CD30-L k.o. mice. Immunization with peptide AA 35 - 55 of the major oligodendrocyte glycoprotein results in experimental allergic encephalitis (EAE) with a 100% incidence in wild type mice of the H2b background and a mean clinical score of 2.5. CD30-L k.o. mice of the same genetic background are resistant to induction of the disease. Since CD30-L can be expressed by activated T cells, by antigen presenting cells (APC) and by epithelial cells (5)(5), the absence of the ligand on APC in tissues may be critical for disease pathogenesis. Alternatively, the absence of the ligand on T cells may cause disruption in fraternal self-regulation; in the latter case the disease could be transferred with T cells from a diseased wild type mouse to a healthy CD30-L knock out recipient.

An elucidation of the function of members of the TNF receptor and ligand family on homeostasis and functional control of T cells will increase the ability to modulate immune response to tumors and other intracellular pathogens that are subverting an effective immune response.

**REFERENCES.**

1. Sallusto, F., Lenig, D., Forster, R., Lipp, M., and Lanzavecchia, A. (1999) Nature 401, 6754, 708
2. Yamazaki, K., Nguyen, T., and Podack, E.R. (1999) J. Immunol. 163, 10, 5178
3. Horie, R. and Watanabe, T. (1998) Semin. Immunol. 10, 6, 457
4. Muta, H., Boise, L.H., Fang, L., and Podack, E.R. (2000) J. Immunol. 165, 9, 5105
5. Romagnani, P., Annunziato, F., Manetti, R., Mavilia, C., Lasagni, L., Manuelli, C., Vannelli, G.B., Vanini, V., Maggi, E., Pupilli, C., and Romagnani, S. (1998) Blood 91, 9, 3323
